# The nm23-H1 gene as a predictor of sensitivity to chemotherapeutic agents in oesophageal squamous cell carcinoma

**DOI:** 10.1038/sj.bjc.6690717

**Published:** 1999-10

**Authors:** N Iizuka, K Hirose, T Noma, S Hazama, A Tangoku, H Hayashi, T Abe, K Yamamoto, M Oka

**Affiliations:** Department of Bioregulatory Function, Department of Surgery II, Department of Biochemistry II, Yamaguchi University School of Medicine, 1144 Kogushi, Ube, Yamaguchi, 755-8505, Japan; Biomedical Research Institute, Kureha Chemical Industry, 3-26-2 Hyakunin-cho, Shinjuku-ku, Tokyo, 169, Japan

**Keywords:** nm23, cisplatin, chemotherapy, prognosis, oesophageal squamous cell carcinoma

## Abstract

Recently, nm23-H1, an anti-metastasis gene, has been reported to correlate with sensitivity to chemotherapeutic agents including cisplatin in human breast and ovarian carcinoma cells. The aim of this study was to evaluate a role for nm23-H1 in responsiveness to cisplatin-based chemotherapy in patients with oesophageal squamous cell carcinoma (OSCC). The expression of nm23-H1 protein was examined immunohistochemically in 32 eligible patients with OSCC who underwent adjuvant chemotherapy with cisplatin, etoposide, and 5-fluorouracil after tumour resection. Fifteen (46.9%) of 32 patients were positive for nm23-H1 staining and 17 (53.1%) were negative. Both disease-free survival and overall survival rates of nm23-H1-negative patients were significantly shorter than in nm23-H1-positive patients (*P* < 0.01 for both). There was no significant difference in clinicopathologic characteristics between nm23-H1-positive and nm23-H1-negative groups. Multivariate analysis also showed that nm23-H1 expression was the most significant factor for overall survival of OSCC patients included in this study (*P* = 0.0007). To further study the role of nm23-H1, a human OSCC cell line (YES-2) was transfected with a plasmid containing a fragment of the nm23-H1 cDNA in an antisense orientation. Reduced expression of nm23-H1 protein in the antisense-transfected (AS) clones was found by Western blot analysis as compared to wild-type YES-2 and YES-2/Neo (clone transfected with the neomycin resistance gene alone). MTT (3-(4,5-dimethyl-2-thiazol)-2,5-diphenyl-2H tetrazolium bromide) assay showed that reduced expression of the nm23-H1 protein in AS clones was consistent with the degree of increased resistance to cisplatin but not etoposide or 5-fluorouracil. These data support the conclusion that reduced expression of nm23-H1 may be associated with resistance to cisplatin, suggesting the value of nm23-H1 expression as a prognostic marker for OSCC patients who are to undergo cisplatin-based chemotherapy. © 1999 Cancer Research Campaign


*British Journal of Cancer* (1999) **81**(3), 469–475
© 1999 Cancer Research Campaign
Article no. bjoc.1999.0717

### The nm23-H1 gene as a predictor of sensitivity to chemotherapeutic agents in oesophageal squamous cell carcinoma

**N Iizuka[1,2], K Hirose[3], T Noma[4], S Hazama[2], A Tangoku[2], H Hayashi[2], T Abe[2], K Yamamoto[2] and M Oka[2]**

[1]Department of Bioregulatory Function, [2]Department of Surgery II and [4]Department of Biochemistry II, Yamaguchi University School of Medicine, 1144 Kogushi, Ube, Yamaguchi 755-8505, Japan; [3]Biomedical Research Institute, Kureha Chemical Industry, 3-26-2 Hyakunin-cho, Shinjuku-ku, Tokyo 169, Japan

**Summary** Recently, nm23-H1, an anti-metastasis gene, has been reported to correlate with sensitivity to chemotherapeutic agents including cisplatin in human breast and ovarian carcinoma cells. The aim of this study was to evaluate a role for nm23-H1 in responsiveness to cisplatin-based chemotherapy in patients with oesophageal squamous cell carcinoma (OSCC). The expression of nm23-H1 protein was examined immunohistochemically in 32 eligible patients with OSCC who underwent adjuvant chemotherapy with cisplatin, etoposide, and 5-fluorouracil after tumour resection. Fifteen (46.9%) of 32 patients were positive for nm23-H1 staining and 17 (53.1%) were negative. Both disease-free survival and overall survival rates of nm23-H1-negative patients were significantly shorter than in nm23-H1-positive patients ($P < 0.01$ for both). There was no significant difference in clinicopathologic characteristics between nm23-H1-positive and nm23-H1-negative groups. Multivariate analysis also showed that nm23-H1 expression was the most significant factor for overall survival of OSCC patients included in this study ($P = 0.0007$). To further study the role of nm23-H1, a human OSCC cell line (YES-2) was transfected with a plasmid containing a fragment of the nm23-H1 cDNA in an antisense orientation. Reduced expression of nm23-H1 protein in the antisense-transfected (AS) clones was found by Western blot analysis as compared to wild-type YES-2 and YES-2/Neo (clone transfected with the neomycin resistance gene alone). MTT (3-(4,5-dimethyl-2-thiazol)-2,5-diphenyl-2H tetrazolium bromide) assay showed that reduced expression of the nm23-H1 protein in AS clones was consistent with the degree of increased resistance to cisplatin but not etoposide or 5-fluorouracil. These data support the conclusion that reduced expression of nm23-H1 may be associated with resistance to cisplatin, suggesting the value of nm23-H1 expression as a prognostic marker for OSCC patients who are to undergo cisplatin-based chemotherapy.
© 1999 Cancer Research Campaign

**Keywords:** nm23; cisplatin; chemotherapy; prognosis; oesophageal squamous cell carcinoma

About half of all patients with oesophageal squamous cell carcinoma (OSCC) have locoregional disease at diagnosis; however, 40–50% of them die of metastatic relapse within the first 2 years after tumour resection (Roder et al, 1994). Thus, the prognosis of patients with locally advanced OSCC remains poor despite significant progress in surgical treatment. Several regimens of cisplatin-based chemotherapy and/or chemoradiotherapy have been applied to treatment of patients with OSCC (Kok et al, 1996; Stahl et al, 1996; Bosset et al, 1997); however, these clinical trials have not shown significant improvement in overall survival so far. These results mean that it may be necessary to distinguish responders from non-responders in cisplatin-based chemotherapy in order to determine a more effective therapy and to improve the poor prognosis of this disease.

The nm23 gene was identified originally as an anti-metastatic influence whose expression was correlated inversely with tumour metastatic potential in murine melanoma cell lines (Steeg et al, 1988). Subsequently, several studies have demonstrated the favourable clinical outcome of overexpression of nm23-H1 in human malignant tumours (Florenes et al, 1992; Tokunaga et al, 1993; Iizuka et al, 1995), although conflicting data have been reported (Higashiyama et al, 1992; Easty et al, 1996; Lindmark et al, 1996). More interestingly, nm23-H1 has been shown to relate to sensitivity to cisplatin in several malignant tumours (Ferguson et al, 1996; Scambia et al, 1996; Freije et al, 1997). Our preliminary study also showed that reduced expression of nm23-H1 protein was associated with poor prognosis of patients with OSCC. However, we observed that there was a lack of association between the expression and prognosis of patients with OSCC who did not undergo cisplatin-based chemotherapy (data not shown). Therefore, in the present study, we focused on a role for nm23-H1 in responsiveness to cisplatin-based chemotherapy in OSCC patients, and we examined nm23-H1 expression in 32 primary tumours of OSCC patients who underwent the same protocol of cisplatin-based chemotherapy following tumour resection. Furthermore, we evaluated the direct correlation between tumour nm23-H1 expression and resistance and/or sensitivity to chemotherapeutic agents including cisplatin using an antisense-transfection assay.

*Received 17 September 1998*
*Revised 1 February 1999*
*Accepted 21 April 1999*

*Correspondence to:* N Iizuka, Department of Bioregulatory Function, Yamaguchi University School of Medicine, Ube, Yamaguchi 755-8505, Japan

### MATERIALS AND METHODS

### Patients

Between April 1989 and July 1996, we had performed cisplatin-based chemotherapy following resection in 35 OSCC patients with stage II–IV defined by the TNM classification of the International Union Against Cancer (1997). Of the 35 patients two died of subarachnoid haemorrhage and myocardial infarction during

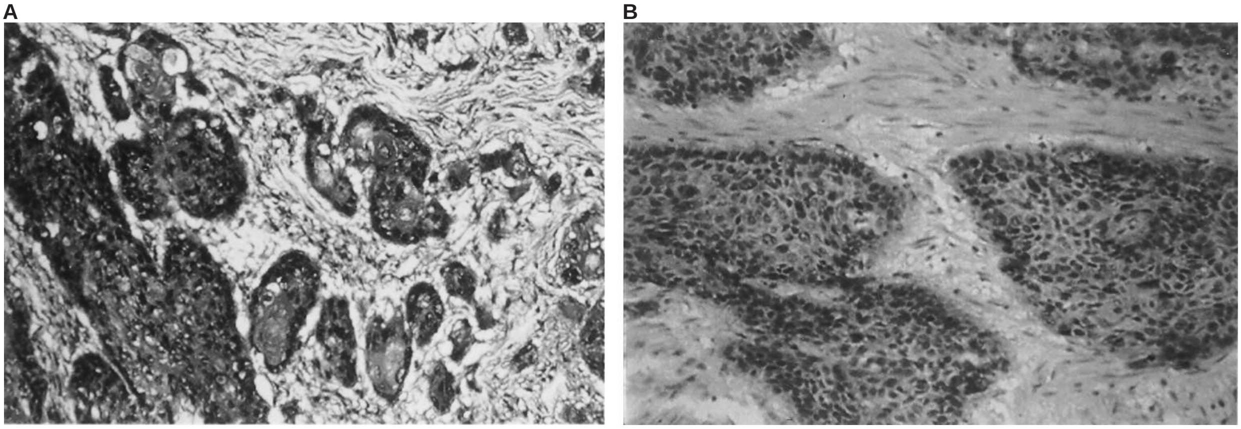

**Figure 1** Immunohistochemical staining for nm23-H1 protein in oesophageal squamous cell carcinoma. (**A**) Representative nm23-H1-positive case which demonstrates intense nm23-H1 immunoreactivity in the cytoplasm of tumour cells (original magnification × 100). (**B**) Representative nm23-H1-negative case in which expression in tumour cells was similar to that of nontumourous tissue (original magnification × 200)

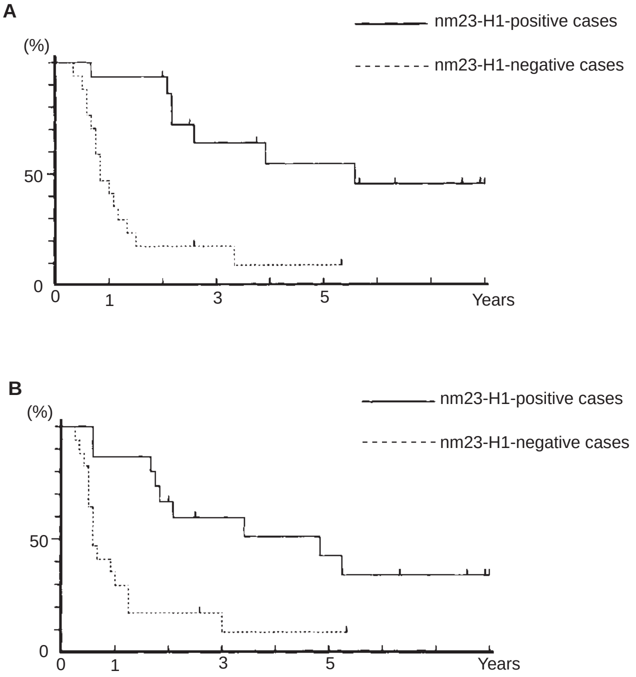

**Figure 2** Overall survival (**A**) and disease-free survival (**B**) curves of patients with OSCC according to nm23-H1 status

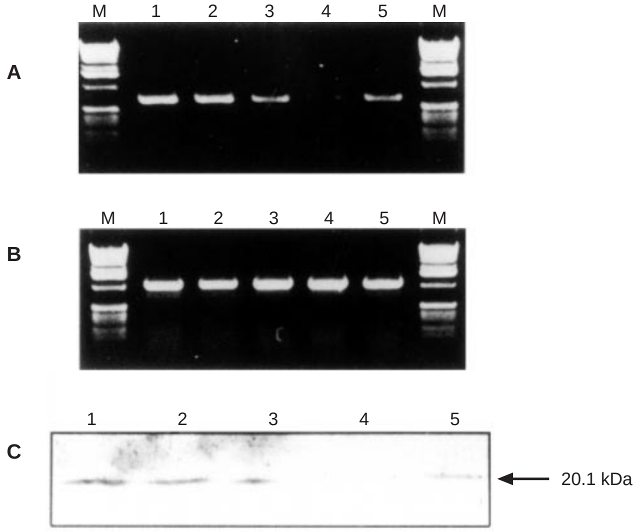

**Figure 3** Down-regulation of both intrinsic mRNA and protein level of nm23-H1 by antisense nm23-H1 cDNA transfection. (**A**) Intrinsic nm23-H1 mRNA expression detected by RT-PCR with primers which yield a 685-bp product and can exclude exogenously transfected antisense nm23-H1 cDNA. (**B**) Expression of β-actin gene (internal control) amplified simultaneously by RT-PCR. (C) Expression of nm23-H1 protein by Western blot analysis with mAb H1-229. Lanes 1–5 are YES-2, YES-2/Neo, YES-2/AS-5, YES-2/AS-12 and YES-2/AS-29 respectively. Relative expression of nm23-H1 protein in YES-2/Neo, YES-2/AS-5, YES-2/AS-12 and YES-2/AS-29 was 0.92, 0.56, 0.04 and 0.41 respectively, when compared to that of YES-2. Lane M is a molecular weight marker, 1 kB DNA ladder (Gibco-BRL, Rockville, MD, USA)

follow-up periods respectively. One patient was excluded because of loss to follow-up. Therefore, the present study was undertaken in 32 patients retrospectively selected. One course of cisplatin-based chemotherapy consisted of etoposide 120 mg $m^{-2}$ per day and 5-fluorouracil (5-FU) 500 mg $m^{-2}$ per day by intravenous (i.v.) continuous infusion on days 3–5, and cisplatin 50 mg $m^{-2}$ per day by i.v. bolus infusion on days 1 and 8. Seven (21.9%) of 32 patients underwent only 1 course of this regimen because of side-effects, while 25 patients (78.1%) underwent two course of this regimen following resection. All patients were subjected to computerized tomography and magnetic resonance imaging every 3 months. These imaging techniques revealed metastatic relapse in 24 (75%) of the 32 patients, and 23 of the 24 patients died of metastatic relapse within the median follow-up period of 65 months (range 21–105 months).

### Immunohistochemical analysis for NM23-H1 protein

The expression of nm23-H1 protein was examined immunohistochemically in 32 primary tumours of 32 eligible patients. Following resection, tumour samples were fixed in 10% formaldehyde solution and embedded in paraffin. Four-micrometre-thick sections were

 © 1999 Cancer Research Campaign

**Table 1** nm23-H1 expression and clinicopathologic characteristics

| Factors | nm23-H1(+) | nm23-H1(–) | *P*-value |
|---|---|---|---|
| Years | | | NS |
| <60 | 7 | 11 | |
| ≥60 | 8 | 6 | |
| Sex | | | NS |
| Male | 13 | 15 | |
| Female | 2 | 2 | |
| Location | | | NS |
| Upper thoracic | 1 | 3 | |
| Mid-thoracic | 11 | 8 | |
| Lower thoracic | 3 | 6 | |
| pT | | | NS |
| pT1 | 0 | 2 | |
| pT2 | 3 | 3 | |
| pT3 | 7 | 7 | |
| pT4 | 5 | 5 | |
| pN | | | NS |
| pN0 | 2 | 1 | |
| pN1 | 13 | 16 | |
| pM | | | NS |
| pM0 | 12 | 12 | |
| pM1 | 3 | 5 | |
| pTNM stage | | | NS |
| II | 4 | 5 | |
| III | 8 | 7 | |
| IV | 3 | 5 | |
| Differentiation | | | NS |
| Well | 2 | 2 | |
| Moderate | 9 | 10 | |
| Poor | 4 | 5 | |
| Venous invasion | | | NS |
| (+) | 9 | 9 | |
| (–) | 6 | 8 | |
| Lymphatic invasion | | | NS |
| (+) | 14 | 13 | |
| (–) | 1 | 4 | |
| Resectability | | | NS |
| R0 | 6 | 5 | |
| R1 | 4 | 7 | |
| R2 | 5 | 5 | |
| EFP therapy | | | NS |
| 1 course | 3 | 4 | |
| 2 course | 12 | 13 | |

NS, not significant; EFP, etoposide, 5-fluorouracil and cisplatin.

deparaffinized in xylene and progressively rehydrated in decreasing concentrations of alcohol. Immunohistochemical staining was performed by the avidin–biotin affinity method with the use of OmniTags (Lipshaw, Pittsburgh, PA, USA). Briefly, the sections were immersed in protein blocking agent for 5 min to reduce non-specific staining and incubated at room temperature for 120 min with 1 μg ml$^{-1}$ anti-human nm23-H1 monoclonal antibody (H1-229, Seikagaku, Tokyo, Japan) (Tokunaga et al, 1993; Iizuka et al, 1995). These sections were washed in chilled PBS three times and were reacted with biotinylated polyvalent antibody at room temperature for 30 min. After being washed in PBS, the sections were reacted with streptavidin–alkaline phosphatase reagent at room temperature for 30 min. Finally, the sections were treated with fast red chromogen for 10 min. Normal mouse IgG was used as a negative control instead of the primary antibodies. The sections were counterstained lightly with haematoxylin. We judged that nm23-H1 protein was positive when more than 20% of the cancer cells were more strongly stained than stromal cells (Figure 1).

### Antisense vector for nm23-H1 cDNA

Nm23-H1 cDNA was isolated by reverse transcription polymerase chain reaction (RT-PCR) using total RNA purified from human peripheral blood mononuclear cells. The primers for nm23-H1 were as follows: sense primer, 5′-GCGTCTAGAGGAACCATGGC-CAACTGTGAGCGT-3′ and antisense primer, 5′-GTCGCGGCC-GCTCTGCCCTCCTGTCATTCATAGAT-3′. The PCR fragment was cloned into a *Not*I/*Xba*I site of the pcDNA II plasmid (Invitrogen, San Diego, CA, USA), and the nucleotide sequence was confirmed by automatic DNA sequencer DSO-1000 (Shimazu, Kyoto, Japan). The same fragment then was recloned into a *Not*I/*Xba*I site of the pRc/CMV eukaryotic expression vector (Invitrogen, San Diego, CA, USA).

### Tumour cells and transfection

YES-2 cells, a human OSCC cell line (Oka et al, 1996; Yamamoto et al, 1997), were maintained in Dulbecco's modified Eagle's medium (Nissui, Tokyo, Japan) supplemented with 5% heat-inactivated fetal serum, 100 U ml$^{-1}$ penicillin G, and 100 μg ml$^{-1}$ streptomycin. Subconfluent cultures in 100 mm Petri dishes were transfected with 5 μg of antisense nm23-H1 expression plasmid or vector alone using the lipofectAMINE reagent (Life Technologies, Rockville, MD, USA) according to the manufacturer's instructions. G418 (600 μg ml$^{-1}$) (Life Technologies, Rockville, MD, USA) was added to the cells 48 h later. G418-resistant clones were isolated and expanded in culture medium containing 200 μg ml$^{-1}$ of G418.

### Effects of antisense transfection on intrinsic nm23-H1 mRNA

The RT-PCR method was performed as previously described (Iizuka et al, 1995). Briefly, cDNA was synthesized from 0.5 μg of total RNA using random hexadeoxynucleotide primers (Takara, Otsu, Japan) and was amplified using AmpliTaq DNA polymerase (Perkin-Elmer/Cetus, San Diego, CA, USA). The primers used to detect only intrinsic nm23-H1 mRNA were 5′-GCAGCCGGAGTTCAAACC-TAA-3′ (sense) and 5′-GCTGGGAGGAAGCATTTTAATCA-3′ (antisense), which yielded a 685-bp product and can exclude exogenously transfected antisense nm23-H1 cDNA. The primers for the β-actin gene were 5′-ATGGATGATGATATCGCCGCGCT-3′ (sense) and 5′-CGGACTCGTCATACTCCTGCTTG-3′ (antisense), which yielded a 1224-bp product. PCR reaction was performed for 27 cycles according to the following parameters: denaturation at 94°C for 1 min, annealing at 55°C for 45 s, extension at 72°C for 2 min. The PCR products were separated electrophoretically on a 1% agarose gel and stained with ethidium bromide.

### Western blot analysis

Western blot analysis was also performed according to previously described method (Iizuka et al, 1995). Briefly, protein samples (50 μg) were subjected to 12.5% sodium dodecyl sulphate polyacrylamide gel electrophoresis (SDS-PAGE) followed by transfer onto nitrocellulose filters. The antibody used was monoclonal antibody (H1-229) which is specifically directed against NM23-H1 protein (Tokunaga et al, 1993). For detection of the immunocomplex, the enhanced chemiluminescence (ECL) Western blotting detection system (Amersham, Tokyo, Japan) was used. Finally, the membrane was exposed to XAR-5 film (Eastman-Kodak, Rochester, NY, USA) for 30 s at room temperature.

© 1999 Cancer Research Campaign 

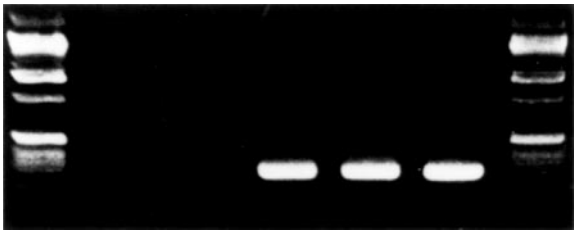

**Figure 4** PCR analysis of genomic DNA extracted from AS transfectants, YES-2/Neo, and YES-2 parental cells for the presence of a plasmid expressing antisense nm23-H1 cDNA fragment. Lanes 1–5 are YES-2, YES-2/Neo, YES-2/AS-5, YES-2/AS-12 and YES-2/AS-29 respectively. Lane M is a molecular weight marker, 1 kB DNA ladder (Gibco-BRL, Rockville, MD, USA)

### Analysis of integration of antisense nm23-H1 cDNA

We used a PCR method to confirm the integration of antisense nm23-H1 cDNA into YES-2 cells by previously reported methods (de Geovani et al, 1996). PCR was performed on genomic DNA extracted by DNAzol (Life Technologies, Rockvile, MD, USA), and 0.5 μg was used for each PCR reaction in 100 μl final volume. The following primers were used to detect antisense nm23-H1 cDNA fragment of the pRc/CMV eukaryotic expression vector: sense primer, 5′-GAGATTATCAAGCGTTTTGAGCA-3′ (position 151–173, exon 2) and antisense primer, 5′-GTATAATGTTC-CTGCCAACTTGT-3′ (position 436–414, exon 4–5), which yielded a 286-bp product. PCR reaction was performed for 30 cycles according to the following parameters: denaturation at 94°C for 30 s, annealing at 55°C for 45 s, extension at 72°C for 90 s.

### Assessment for in vitro cytotoxity induced by chemotherapeutic agents

Cisplatin and etoposide were purchased from Nippon Kayaku (Tokyo, Japan), whereas 5-FU was obtained from Kyowa Hakko (Tokyo, Japan). MTT (3-(4,5-dimethyl-2-thiazol)-2,5-diphenyl-2H tetrazolium bromide; Dojindo, Tokyo, Japan) assay was performed to evaluate the in vitro cytotoxity induced by cisplatin, etoposide and 5-FU in YES-2, YES-2/Neo, and AS clones. The cells were plated at $5 \times 10^3$ per 100 μl in 96-well plates in Dulbecco's modified Eagle's medium with 5% fetal calf serum, and allowed to attach overnight. Three wells were treated with each drug concentration of 0.01 μg $ml^{-1}$ to 20 μg $ml^{-1}$ of cisplatin or etoposide or 0.01 μg $ml^{-1}$ to 50 μg $ml^{-1}$ of 5-FU. After 48 h, cell viability was calculated as the percentage of control cultures which were not exposed to drugs. Thus, the experiments were performed in triplicate and the results were evaluated using the means of three experiments.

### Statistical analysis

The $\chi^2$ test with Yates' correction or Fisher's exact test was used to elucidate the correlation between nm23-H1 expression and clinicopathological characteristics. All survival and disease-free survival data were analysed by the Kaplan–Meier method using Cox–Mantel comparisons for statistical significance (Kaplan and Meier, 1958; Cox, 1972). Multivariate analysis by the Cox proportional hazards regression model was performed to evaluate the factors responsible for overall survival of OSCC patients who underwent cisplatin-based chemotherapy after tumour resection. Pearson's correlation test was used to analyse the relationship between nm23-H1 protein level and $ID_{50}$ of cisplatin in YES-2 and the antisense transfectants.

**Table 2** Factors linked to overall survival

| Factors coefficient | Regression error | Standard | *t*-value | *P*-value |
|---|---|---|---|---|
| Nm23-H1 staining | 2.1023 | 0.5549 | 3.789 | 0.0007 |
| Sex | 1.9963 | 0.6733 | 2.920 | 0.0068 |
| Tumour location | 0.3590 | 0.3091 | 1.162 | 0.2552 |

**Table 3** In vitro inhibitory effect of cisplatin on control- and antisense nm23-H1 transfected tumour cell lines

| Cell line | nm23-H1 protein level | $ID_{50}$ (μg $ml^{-1}$) |
|---|---|---|
| YES-2 | 1.00 | 1.3 |
| YES-2/Neo | 0.92 | 1.1 |
| YES-2/AS-5 | 0.56 | 2.1 |
| YES-2/AS-12 | 0.04 | 4.1 |
| YES-2/AS-29 | 0.41 | 2.0 |

$ID^{50}$, the cisplatin dose at which the cell number is 50% of control (untreated) cultures as determined by MTT assay.

### RESULTS

### Immunohistochemical study

Of 32 patients included in this study, 15 (46.9%) were positive for nm23-H1 staining and 17 (53.1%) were negative. In 15 (88.2%) of 17 nm23-H1-negative cases, the intensity of nm23-H1 immunostaining of the primary tumour was weaker than corresponding normal oesophageal epithelium (data not shown). The disease-free survival and overall survival curves for patients with positive or negative nm23-H1 staining are shown in Figure 2. Overall 1-, 3- and 5-year survival rates of nm23-H1-negative patients were 41.2%, 17.6% and 8.8% respectively. By contrast, those of nm23-H1-positive patients were 93.3%, 63.8% and 54.7% respectively. Disease-free 1-, 3- and 5-year survival rates of nm23-H1-negative patients were 29.4%, 8.8% and 8.8% respectively, while those of nm23-H1-positive patients were 86.7%, 59.3% and 42.3% respectively. Thus, nm23-H1-negative patients had both significantly shorter disease-free survival and overall survival than did nm23-H1-positive patients (Figure 2, $P < 0.01$ for both). Multivariate analysis also showed that, of ten factors examined, nm23-H1 expression was the most significant factor for overall survival of OSCC patients who underwent cisplatin-based chemotherapy after tumour resection ($P = 0.0007$, Table 1). Of the ten OSCC patients who had poor resectability (R2 resection), two (40%) of five patients with positive nm23-H1 staining are alive in condition of relapse-free after this chemotherapy, on the contrary, all of the five patients with negative nm23-H1 staining died of metastatic relapse. As shown in Table 2, there was no significant difference in the other clinicopathological characteristics between nm23-H1-positive and nm23-H1-negative groups. No association was observed between nm23-H1 expression and local immune response such as lymphocyte infiltration (data not shown).

 *© 1999 Cancer Research Campaign*

### In vitro cytotoxic effect of chemotherapeutic agents on control- and antisense nm23-H1-transfected cells

Transfection of antisense nm23-H1 cDNA was performed to evaluate the relation of nm23-H1 to cytotoxity induced by chemotherapeutic agents in OSCC cells. Of 30 G418-resistant clones isolated in culture medium containing 600 μg ml$^{-1}$ of G418, our RT-PCR method selected these clones, YES-2/AS-5, YES-2/AS-12 and YES-2/AS-29, which showed reduced expression of intrinsic nm23-H1 mRNA (Figure 3). Subsequently, the nm23-H1 protein level in these clones was analysed by Western blot analysis (Figure 3). Relative expression of nm23-H1 protein in YES-2/AS-5, YES-2/AS-12, YES-2/AS-29 and YES-2/Neo calculated by densitometer was 0.56, 0.04, 0.41 and 0.92 respectively, when compared to that of YES-2 cells (parental cell). Furthermore, the integration of antisense nm23-H1 cDNA into YES-2/AS-5, YES-2/AS-12 and YES-2/AS-29 was confirmed by PCR analysis with genomic DNA extracted from these cells (Figure 4). Namely, a spliced 286-bp product was amplified from genomic DNA of the AS clones as well as from a plasmid DNA, but not from YES-2 and YES-2/Neo cells.

We used MTT assay in order to evaluate in vitro cytotoxicity induced by cisplatin, etoposide and 5-FU. There was no difference in growth rates between the AS clones and YES-2 or YES-2/Neo (data not shown). Increased resistance of the AS clones to cisplatin was observed when compared to YES-2 or YES-2/Neo (Figure 5). The $ID_{50}$ of YES-2/AS-5, YES-2/AS-12, YES-2/AS-29 and YES-2/Neo for cisplatin treatment was 2.1, 4.1, 2.0 and 1.1 μg ml$^{-1}$ respectively. A reduction of twofold in the expression level of nm23-H1 by YES-2/AS-5 and YES-2/AS-29 resulted in twofold increased resistance to cisplatin as compared to YES-2/Neo. Furthermore, a reduction of 23-fold in the expression level of nm23-H1 by YES-2/AS-12 resulted in fourfold increased resistance to cisplatin as compared to YES-2/Neo (Table 3). Thus, nm23-H1 protein level in the five cells including the parental cell was associated inversely with $ID_{50}$ of CDDP ($r = -0.935$, $P < 0.02$). By contrast, there was no difference in resistance to etoposide or 5-FU between AS clones and YES-2/Neo, although YES-2/AS-12, with markedly reduced expression of nm23-H1 protein, showed increased resistance to 5-FU (Figure 5).

### DISCUSSION

Cisplatin is one of the key drugs for treatment of patients with OSCC (Kok et al, 1996). However, cisplatin-based chemotherapy dose not sufficiently improve their overall survival, even when combined with additional irradiation (Stahl et al, 1996; Bosset et al, 1997). The results of these clinical trials suggest that it might be necessary to select the most effective therapy for each case in order to improve survival of patients with OSCC, especially advanced OSCC. Recent studies have elucidated several molecules, such as p53, GST-π and metalothionein, responsible for sensitivity to cisplatin (Timmer-Bosscha et al, 1993; Rusch et al, 1995; Hishikawa et al, 1997). More recently, nm23-H1 has been reported to correlate directly with sensitivity to cisplatin as well as other alkylating agents by transfection assay (Ferguson et al, 1996) and the expression of nm23-H1 protein has been shown to correlate inversely with prognosis of patients with ovarian carcinoma after cisplatin-based chemotherapy (Scambia et al, 1996).

With regard to OSCC, work by Patel et al (1997) has shown that reduced expression of nm23 protein was a factor associated with poor prognosis of OSCC patients after resection; however, they did not refer to the relation of nm23 expression to drug sensitivity.

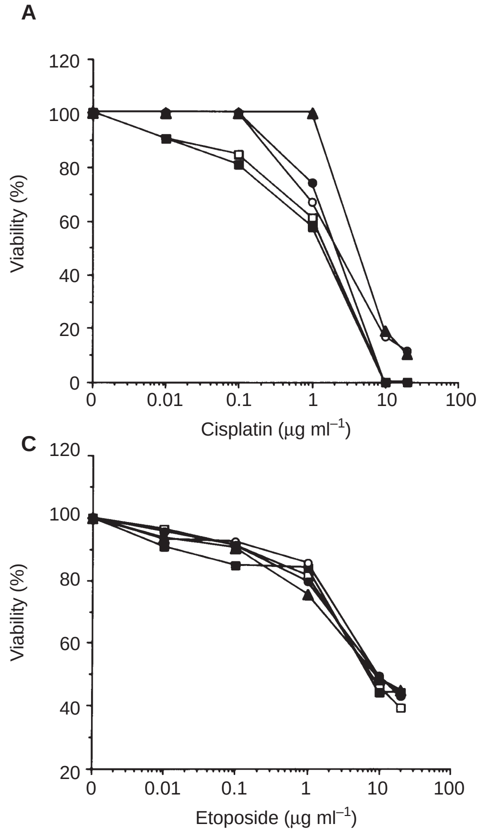

**Figure 5** Dose–response of parental YES-2 (□), YES-2/Neo (■), and AS transfectants (YES-2/AS-5, YES-2/AS-12 and YES-2/AS-29, ○, ▲ and ● respectively) to cisplatin (**A**), 5-fluorouracil (**B**) and etoposide (**C**). Cells ($5 \times 10^3$) were plated with semilog doses of cisplatin or etoposide ranging from 10 ng ml$^{-1}$ to 20 μg ml$^{-1}$ and 5-fluorouracil ranging from 10 ng ml$^{-1}$ to 50 μg ml$^{-1}$. On day 2 of culture, the number of viable cells per well was determined by MTT assay. The experiments were performed in triplicate and the mean value was plotted

© 1999 Cancer Research Campaign

Our immunohistochemical study showed that expression of nm23-H1 protein was correlated inversely with disease-free survival and overall survival rates of OSCC patients who underwent cisplatin-based chemotherapy following resection. Multivariate analysis showed that nm23-H1 status was most responsible for overall survival of OSCC patients after cisplatin-based chemotherapy. Furthermore, we confirmed that, of the ten OSCC patients who had poor resectability (R2 resection), two (40%) of five patients with positive nm23-H1 staining were alive and relapse free after this chemotherapy, on the contrary, all of the five patients with negative nm23-H1 staining died of metastatic relapse. Thus, nm23-H1 is likely to relate to responsiveness to cisplatin-based chemotherapy in patients with OSCC. Stahl et al (1996) have reported that overall 3-year survival rate was 33% in patients with T2–4 oesophageal carcinoma who underwent preoperative chemoradiotherapy and surgery. Likewise, we found that overall 3-year survival rate of 32 OSCC patients included in this study was 39.6%. However, that of 15 OSCC patients with positive nm23-H1 staining was 63.8% after this chemotherapy. These results suggest that nm23-H1 status may be a useful marker for cisplatin-based chemotherapy in patients with OSCC.

MTT assay showed enhanced resistance to cisplatin, but not etoposide or 5-FU, in parallel to the degree of reduction of nm23-H1 protein level in the OSCC cells examined. This result was most consistent with the data reported by Freije et al (1997), except for 5-FU. The role of nm23-H1 in cisplatin-induced cytotoxicity remains to be clarified. Ferguson et al (1996) have demonstrated increased formation of interstrand DNA cross-links in the nm23-H1 transfectants showing overexpression of nm23-H1, and there was no difference in the rates of DNA repair. Since it also was confirmed that the nm23-H1 level was increased in S phase (Caligo et al, 1995), and cisplatin has the strongest cytotoxic effect in S-G2 phase (Nguyen et al, 1993), one possibility is that nm23-H1 may play a role in cisplatin-induced cytotoxity via modulating the cell cycle. However, our data with flow cytometry demonstrated that there was no difference in the cell cycle between parental YES-2 and YES-2/Neo and YES-2/AS-12 (data not shown). Recent studies have shown that mitochondria play an important role in the cytotoxicity or apoptosis induced by cisplatin, but not 5-FU or etoposide (Andrews and Albright, 1992; Olivero et al, 1995). The MTT assay is considered to be a representative mitochondrial function assay (Berridge and Tan, 1993); therefore, it is possible that nm23-H1 may be directly related to mitochondrial dysfunction induced by cisplatin in OSCC. This concept is supported by a previous report that respiration could be aided by ADP formed by adenylate kinase or nm23/NDP kinase in mitochondria (Gauthier et al, 1990). Further studies are required to clarify the roles of nm23-H1/NDP kinase A in mitochondrial dysfunction and/or apoptosis induced by cisplatin in human OSCC cells.

There were no differences in resistance to 5-FU between YES-2/AS-5 and YES-2/AS-29 and YES-2/Neo; however, YES-2/AS-12, with markedly reduced expression of nm23-H1 protein, showed increased resistance to 5-FU. The reason why only YES-2/AS-12 showed increased resistance to 5-FU remain unknown. It has been reported that several molecules such as Bcl-2 family proteins and dihydropyrimidine dehydrogenase contributed to the sensitivity to 5-FU (McLeod et al, 1998; Nita et al, 1998). Current study is attempting to clarify the relation or the interaction between nm23-H1 and these molecules in YES-2/AS-12.

In conclusion, the close relation of nm23-H1 status with resistance to cisplatin suggests that it may be a useful marker for cisplatin-based chemotherapy in patients with OSCC. More interestingly, gamma linoleic acid, n-6 polyunsaturated fatty acid, was reported to increase the expression of nm23-H1 in human cancer cells (Jiang et al, 1998). Thus, modulating tumour nm23-H1 expression may be considered as a potential therapeutic strategy in combination with cisplatin treatment in OSCC patients.

### ACKNOWLEDGEMENT

This work was supported in part by a Grant-in-Aid from the Ministry of Education, Science, Sports and Culture of Japan.

### REFERENCES

Andrews PA and Albright KD (1992) Mitochondrial defects in cis-diamminedichloroplatinum (II)-resistant human ovarian carcinoma cells. *Cancer Res* **52**: 1895–1901

Berridge MV and Tan AS (1993) Characterization of the cellular reduction of 3-(4,5-dimethylthiazol-2-yl)-2,5-diphenyltetrazolium bromide (MTT): subcellular localization, substrate dependence, and involvement of mitochondrial electron transport in MTT reduction. *Arch Biochem Biophys* **303**: 474–482

Bosset JF, Gignoux M, Triboulet JP, Tiret E, Mantion G, Elias D, Lozach P, Ollier J, Pavy JJ, Mercier M and Sahmoud T (1997) Chemoradiotherapy followed by surgery compared with surgery alone in squamous-cell cancer of the esophagus. *N Engl J Med* **337**: 161–167

Caligo MA, Cipollini G, Fiore L, Calvo S, Basolo F, Collecchi P, Ciardiello F, Pepe S, Petrini M and Bevilacqua G (1995) NM23 gene expression correlates with cell growth rate and S-phase. *Int J Cancer* **60**: 837–842

Cox DR (1972) Regression models and life table. *J R Stat Soc* **34**: 187–220

de Geovani C, Landuzzi L, Frabetti F, Nicoletti G, Griffoni C, Rossi I, Mazzotti M, Scotto L, Nanni P and Lollini PL (1996) Antisense epidermal growth factor receptor transfection impairs the proliferative ability of human rhabdomyosarcoma cells. *Cancer Res* **56**: 3898–3901

Easty DJ, Maung K, Lascu I, Veron M, Fallowfield ME, Hart IR and Bennett DC (1996) Expression of NM23 in human melanoma progression and metastasis. *Br J Cancer* **74**: 109–114

Ferguson AW, Flatow U, MacDonald NJ, Larminat F, Bohr VA and Steeg PS (1996) Increased sensitivity to cisplatin by nm23-transfected tumor cell lines. *Cancer Res* **56**: 2931–2935

Florenes VA, Aamdal S, Myklebost O, Maelandsmo GM, Bruland OS and Fodstad O (1992) Levels of nm23 messenger RNA in metastatic malignant melanomas: inverse correlation to disease progression. *Cancer Res* **52**: 6088–6091

Freije JM, Lawrence JA, Hollingshead MG, De la Rosa A, Narayanan V, Grever M, Sausville EA, Paull K and Steeg PS (1997) Identification of compounds with preferential inhibitory activity against low-Nm23-expressing human breast carcinoma and melanoma cell lines. *Nat Med* **4**: 395–401

Gauthier T, Denis-Pouxviel C and Murat JC (1990) Respiration of mitochondria isolated from differentiated and undifferentiated HT29 colon cancer cells in the presence of various substrates and ADP generating systems. *Int J Biochem* **22**: 411–417

Higashiyama M, Doi O, Yokouchi H, Kodama K, Nakamori S, Tateishi R and Kimura N (1992) Immunohistochemical analysis of nm23 gene product/NDP kinase expression in pulmonary adenocarcinoma: Lack of prognostic value. *Br J Cancer* **66**: 533–536

Hishikawa Y, Abe S, Kinugasa S, Yoshimura H, Monden N, Igarashi M, Tachibana M and Nagasue N (1997) Overexpression of metallothionein correlates with chemoresistance to cisplatin and prognosis in esophageal cancer. *Oncology* **54**: 342–347

Iizuka N, Oka M, Noma T, Nakazawa A, Hirose K and Suzuki T (1995) NM23-H1 and NM23-H2 messenger RNA abundance in human hepatocellular carcinoma. *Cancer Res* **55**: 652–657

International Union Against Cancer. Sobin LH and Wittekind C (1997) *TNM Classification of Malignant Tumors*, 5th edn, pp. 54–58. Wiley-Liss Inc: New York

Jiang WG, Hiscox S, Bryce RP, Horrobin DF and Mansel RE (1998) The effects of n-6 polyunsaturated fatty acids on the expression of nm-23 in human cancer cells. *Br J Cancer* **77**: 731–738

Kaplan BL and Meier P (1958) Nonparametic estimation from incomplete observations. *J Am Stat Assoc* **53**: 457–481

 © 1999 Cancer Research Campaign

Kok TC, Van der Gaast A, Dees J, Eykenboom WM, Van Overhagen H, Stoter G, Tilanus HW and Splinter TA (1996) Cisplatin and etoposide in oesophageal cancer: a phase II study. *Br J Cancer* **74**: 980–984

Lindmark G (1996) NM-23 H1 immunohistochemistry is not useful as predictor of metastatic potential of colorectal cancer. *Br J Cancer* **74**: 1413–1418

McLeod HL, Sludden J, Murray GI, Keenan RA, Davidson AI, Park K, Koruth M and Cassidy J (1998) Characterization of dihydropyrimidine dehydrogenase in human colorectal tumours. *Br J Cancer* **77**: 461–465

Nguyen HN, Sevin BU, Averette HE, Perras J, Ramos R, Donato D, Ochiai K and Penalver M (1993) Cell cycle perturbations of platinum derivatives on two ovarian cancer cell lines. *Cancer Invest* **11**: 264–275

Nita ME, Nagawa H, Tominaga O, Tsuno N, Fujii S, Sasaki S, Fu CG, Takenoue T, Tsuruo T and Muto T (1998) 5-Fluorouracil induces apoptosis in human colon cancer cell lines with modulation of Bcl-2 family proteins. *Br J Cancer* **78**: 986–992

Oka M, Iizuka N, Yamamoto K, Gondo T, Abe T, Hazama S, Akitomi Y, Koshihara Y, Ohsugi Y, Ooba Y, Ishihara T and Suzuki T (1996) The influence of interleukin-6 on the growth of human esophageal cancer cell lines. *J Interferon Cytokine Res* **16**: 1001–1006

Olivero OA, Semino C, Kassim A, Lopez-Larraza DM and Poirier MC (1995) Preferential binding of cisplatin to mitochondrial DNA of Chinese hamster ovary cells. *Mutation Res* **346**: 221–230

Patel DD, Bhatavdekar JM, Chikhlikar PR, Patel YV, Shah NG, Ghosh N, Suthar TP and Balbar DB (1997) Clinical significance of p53, nm23, and bcl-2 in T3–4N1M0 oesophageal carcinoma: an immunohistochemical approach. *J Surg Oncol* **65**: 111–116

Roder JD, Busch R, Stein HJ, Fink U and Siewert JR (1994) Ratio of invaded to removed lymph nodes as a predictor of survival in squamous cell carcinoma of the oesophagus. *Br J Surg* **81**: 410–413

Rusch V, Klimstra D, Venkatraman E, Oliver J, Martini N, Gralla R, Kris M and Dmitrovsky E (1995) Aberrant p53 expression predicts clinical resistance to cisplatin-based chemotherapy in locally advanced non-small cell lung cancer. *Cancer Res* **55**: 5038–5042

Scambia G, Ferrandina G, Marone M, Benedetti PP, Giannitelli C, Piantelli M, Leone A and Mancuso S (1996) Nm23 in ovarian cancer: correlation with clinical outcome and other clinicopathologic and biochemical prognostic parameters. *J Clin Oncol* **14**: 334–342

Stahl M, Wilke H, Fink U, Stuschke M, Walz MK, Siewert JR, Moll M, Fett W, Makoski HB, Breuer N, Schmidt U, Niebel W, Sack H, Eigler FW and Seeber S (1996) Combined preoperative chemotherapy and radiotherapy in patients with locally advanced esophageal cancer. Interim analysis of a phase II trial. *J Clin Oncol* **14**: 829–837

Steeg PS, Bevilacqua G, Kopper L, Thorgeirsson UP, Talmadge JB, Liotta LA and Sobel M (1988) Evidence for a novel gene associated with low tumor metastatic potential. *J Natl Cancer Inst* **80**: 200–204

Timmer-Bosscha H, Timmer A, Meijer C, de Vries EG, de Jong B, Oosterhuis JW and Mulder NH (1993) cis-diamminedichloroplatinum(ii) resistance in vitro and in vivo in human embryonal carcinoma cells. *Cancer Res* **53**: 5707–5713

Tokunaga Y, Urano T, Furukawa K, Kondo H, Kanematsu T and Shiku H (1993) Reduced expression of nm23-H1, but not of nm23-H2 is concordant with the frequency of lymph node metastasis of human breast cancer. *Int J Cancer* **55**: 66–71

Yamamoto K, Oka M, Hayashi H, Tangoku A, Gondo T and Suzuki T (1997) CYFRA 21-1 is a useful marker for esophageal squamous cell carcinoma. *Cancer* **79**: 1647–1655

*© 1999 Cancer Research Campaign*

## References

[PDF_00570] Andrews P. A., Albright K. D. (1992). Mitochondrial defects in cis-diamminedichloroplatinum(II)-resistant human ovarian carcinoma cells.. Cancer Res.

[PDF_00573] Berridge M. V., Tan A. S. (1993). Characterization of the cellular reduction of 3-(4,5-dimethylthiazol-2-yl)-2,5-diphenyltetrazolium bromide (MTT): subcellular localization, substrate dependence, and involvement of mitochondrial electron transport in MTT reduction.. Arch Biochem Biophys.

[PDF_00577] Bosset J. F., Gignoux M., Triboulet J. P., Tiret E., Mantion G., Elias D., Lozach P., Ollier J. C., Pavy J. J., Mercier M. (1997). Chemoradiotherapy followed by surgery compared with surgery alone in squamous-cell cancer of the esophagus.. N Engl J Med.

[PDF_00581] Caligo M. A., Cipollini G., Fiore L., Calvo S., Basolo F., Collecchi P., Ciardiello F., Pepe S., Petrini M., Bevilacqua G. (1995). NM23 gene expression correlates with cell growth rate and S-phase.. Int J Cancer.

[PDF_00585] De Giovanni C., Landuzzi L., Frabetti F., Nicoletti G., Griffoni C., Rossi I., Mazzotti M., Scotto L., Nanni P., Lollini P. L. (1996). Antisense epidermal growth factor receptor transfection impairs the proliferative ability of human rhabdomyosarcoma cells.. Cancer Res.

[PDF_00589] Easty D. J., Maung K., Lascu I., Véron M., Fallowfield M. E., Hart I. R., Bennett D. C. (1996). Expression of NM23 in human melanoma progression and metastasis.. Br J Cancer.

[PDF_00592] Ferguson A. W., Flatow U., MacDonald N. J., Larminat F., Bohr V. A., Steeg P. S. (1996). Increased sensitivity to cisplatin by nm23-transfected tumor cell lines.. Cancer Res.

[PDF_00595] Flørenes V. A., Aamdal S., Myklebost O., Maelandsmo G. M., Bruland O. S., Fodstad O. (1992). Levels of nm23 messenger RNA in metastatic malignant melanomas: inverse correlation to disease progression.. Cancer Res.

[PDF_00598] Freije J. M., Lawrence J. A., Hollingshead M. G., De la Rosa A., Narayanan V., Grever M., Sausville E. A., Paull K., Steeg P. S. (1997). Identification of compounds with preferential inhibitory activity against low-Nm23-expressing human breast carcinoma and melanoma cell lines.. Nat Med.

[PDF_00602] Gauthier T., Denis-Pouxviel C., Murat J. C. (1990). Respiration of mitochondria isolated from differentiated and undifferentiated HT29 colon cancer cells in the presence of various substrates and ADP generating systems.. Int J Biochem.

[PDF_00606] Higashiyama M., Doi O., Yokouchi H., Kodama K., Nakamori S., Tateishi R., Kimura N. (1992). Immunohistochemical analysis of nm23 gene product/NDP kinase expression in pulmonary adenocarcinoma: lack of prognostic value.. Br J Cancer.

[PDF_00610] Hishikawa Y., Abe S., Kinugasa S., Yoshimura H., Monden N., Igarashi M., Tachibana M., Nagasue N. (1997). Overexpression of metallothionein correlates with chemoresistance to cisplatin and prognosis in esophageal cancer.. Oncology.

[PDF_00614] Iizuka N., Oka M., Noma T., Nakazawa A., Hirose K., Suzuki T. (1995). NM23-H1 and NM23-H2 messenger RNA abundance in human hepatocellular carcinoma.. Cancer Res.

[PDF_00620] Jiang W. G., Hiscox S., Bryce R. P., Horrobin D. F., Mansel R. E. (1998). The effects of n-6 polyunsaturated fatty acids on the expression of nm-23 in human cancer cells.. Br J Cancer.

[PDF_00627] Kok T. C., Van der Gaast A., Dees J., Eykenboom W. M., Van Overhagen H., Stoter G., Tilanus H. W., Splinter T. A. (1996). Cisplatin and etoposide in oesophageal cancer: a phase II study. Rotterdam Oesophageal Tumour Study Group.. Br J Cancer.

[PDF_00630] Lindmark G. (1996). NM-23 H1 immunohistochemistry is not useful as predictor of metastatic potential of colorectal cancer.. Br J Cancer.

[PDF_00632] McLeod H. L., Sludden J., Murray G. I., Keenan R. A., Davidson A. I., Park K., Koruth M., Cassidy J. (1998). Characterization of dihydropyrimidine dehydrogenase in human colorectal tumours.. Br J Cancer.

[PDF_00635] Nguyen H. N., Sevin B. U., Averette H. E., Perras J., Ramos R., Donato D., Ochiai K., Penalver M. (1993). Cell cycle perturbations of platinum derivatives on two ovarian cancer cell lines.. Cancer Invest.

[PDF_00638] Nita M. E., Nagawa H., Tominaga O., Tsuno N., Fujii S., Sasaki S., Fu C. G., Takenoue T., Tsuruo T., Muto T. (1998). 5-Fluorouracil induces apoptosis in human colon cancer cell lines with modulation of Bcl-2 family proteins.. Br J Cancer.

[PDF_00642] Oka M., Iizuka N., Yamamoto K., Gondo T., Abe T., Hazama S., Akitomi Y., Koishihara Y., Ohsugi Y., Ooba Y. (1996). The influence of interleukin-6 on the growth of human esophageal cancer cell lines.. J Interferon Cytokine Res.

[PDF_00646] Olivero O. A., Semino C., Kassim A., Lopez-Larraza D. M., Poirier M. C. (1995). Preferential binding of cisplatin to mitochondrial DNA of Chinese hamster ovary cells.. Mutat Res.

[PDF_00649] Patel D. D., Bhatavdekar J. M., Chikhlikar P. R., Patel Y. V., Shah N. G., Ghosh N., Suthar T. P., Balar D. B. (1997). Clinical significance of p53, nm23, and bcl-2 in T3-4N1M0 oesophageal carcinoma: an immunohistochemical approach.. J Surg Oncol.

[PDF_00653] Roder J. D., Busch R., Stein H. J., Fink U., Siewert J. R. (1994). Ratio of invaded to removed lymph nodes as a predictor of survival in squamous cell carcinoma of the oesophagus.. Br J Surg.

[PDF_00656] Rusch V., Klimstra D., Venkatraman E., Oliver J., Martini N., Gralla R., Kris M., Dmitrovsky E. (1995). Aberrant p53 expression predicts clinical resistance to cisplatin-based chemotherapy in locally advanced non-small cell lung cancer.. Cancer Res.

[PDF_00660] Scambia G., Ferrandina G., Marone M., Benedetti Panici P., Giannitelli C., Piantelli M., Leone A., Mancuso S. (1996). nm23 in ovarian cancer: correlation with clinical outcome and other clinicopathologic and biochemical prognostic parameters.. J Clin Oncol.

[PDF_00664] Stahl M., Wilke H., Fink U., Stuschke M., Walz M. K., Siewert J. R., Molls M., Fett W., Makoski H. B., Breuer N. (1996). Combined preoperative chemotherapy and radiotherapy in patients with locally advanced esophageal cancer. Interim analysis of a phase II trial.. J Clin Oncol.

[PDF_00669] Steeg P. S., Bevilacqua G., Kopper L., Thorgeirsson U. P., Talmadge J. E., Liotta L. A., Sobel M. E. (1988). Evidence for a novel gene associated with low tumor metastatic potential.. J Natl Cancer Inst.

[PDF_00672] Timmer-Bosscha H., Timmer A., Meijer C., de Vries E. G., de Jong B., Oosterhuis J. W., Mulder N. H. (1993). cis-diamminedichloroplatinum(ii) resistance in vitro and in vivo in human embryonal carcinoma cells.. Cancer Res.

[PDF_00675] Tokunaga Y., Urano T., Furukawa K., Kondo H., Kanematsu T., Shiku H. (1993). Reduced expression of nm23-H1, but not of nm23-H2, is concordant with the frequency of lymph-node metastasis of human breast cancer.. Int J Cancer.

[PDF_00679] Yamamoto K., Oka M., Hayashi H., Tangoku A., Gondo T., Suzuki T. (1997). CYFRA 21-1 is a useful marker for esophageal squamous cell carcinoma.. Cancer.

